# Characterization and phylogenetic analysis of the complete chloroplast genome of *Tulipa patens* (Liliaceae)

**DOI:** 10.1080/23802359.2021.1967799

**Published:** 2021-08-25

**Authors:** Xiuting Ju, Guomin Shi, Shengrong Chen, Wubin Dai, Tao He

**Affiliations:** a College of Agriculture and Animal Husbandry, Qinghai University, Xining, China; b The Key Laboratory of Landscape Plants of Qinghai Province, Xining, China; c State Key Laboratory of Plateau Ecology and Agriculture, Qinghai University, Xining, China; d The Academic Administration Office of Qinghai University, Xining, China; e College of Ecological Environment Engineering, Qinghai University, Xining, China

**Keywords:** *Tulipa patens*, Liliaceae, chloroplast genome

## Abstract

The chloroplast genome and evolutionary relationship analysis of *Tulipa patens* could provide fundamental genetic reference for its molecular breeding and biological research. The complete chloroplast genome of *T. patens* was sequenced and reported here. The genome was 152,050 bp in length, containing a pair of inverted repeated regions (26,330 bp) which were separated by a large single copy region of 82,184 bp, and a small single copy region of 17,206 bp. A total of 133 functional genes were annotated, including 87 protein-coding genes, 38 tRNA genes, and eight rRNA genes. The phylogenetic relationships of 10 species indicated that *T. patens* was closely related to *Tulipa sylvestris*.

The Liliaceae family includes approximately 250 genera and 3500 species distributed worldwide. Tulips (genus *Tulipa* and family Liliaceae) are among the world's most well-known, beloved, and economically important ornamental plants (Li et al. 2017) and a variety of cultivars are grown for cut flowers, flowering potted plants, and landscaping (Van Tuyl and van Creij 2007). There are about 150 species of *Tulipa*, mainly distributed in Asia, Europe and North Africa, among which the Mediterranean to Central Asia is the most abundant (Xing et al. 2017), constituting a great genetic resource for the continued breeding and improvement of tulip cultivars (Xing et al. 2020). Chloroplasts are organelles that are essential for photosynthesis (Mehmood et al. 2020). The structure and composition of plastid genomes have become widely utilized in identifying unique genetic changes and evolutionary relationships of various groups of plants (Mehmood et al. 2020). Here, we sequenced the complete chloroplast genome of *Tulipa patens* (Genbank accession number: MT327740), to provide more primary genetic information for the phylogenetic relationship analysis of the genus and other relevant research.

In this study, *T. patens* were collected from Altay Region, Buerjing County, Xinjiang Province, China (48°41′48″ N, 87°02′03″ E) in September 2018. A specimen was deposited at Key Laboratory of Landscape Plants of Qinghai Province, Qinghai University, Xining, China (Xiu-Ting, JU, juxiuting@163.com) under the voucher number JXT-2019-YJX018. The total genomic DNA was extracted using leaves from the same plant. After DNA extraction, a library with an insertion size of 350 bp was constructed, and genomic sequencing was performed on the Illumina HiSeq Platform (Illumina, San Diego, CA) with a read length of 150 bp. The software SPAdes v.3.10.1 (Bankevich, et al. 2012) was employed to assemble the chloroplast genome, kmer uses 55, 87,121. Then, Prodigalv2.6.3 (https://www.github.com/hyattpd/Prodigal) was used to annotate the coding sequences (CDs) under the default parameter settings. Hmmer v3.1b2 (http://www.hmmer.org/) and Aragorn v1.2.38 (http://130.235.244.92/ARAGORN/) were respectively used to predict transfer RNA (tRNA) genes and ribosomal RNA (rRNA) genes under the default parameter settings.

The complete chloroplast genome of *T. patens* was 152,050 bp in length with a typical quadripartite structure, containing a pair of inverted repeated (IR) regions (26,330 bp) that were separated by a large single copy (LSC) region of 82,184 bp, and a small single copy (SSC) region of 17,206 bp. The average coverage depth of the assembly was 1876.2875. The GC content of the whole complete chloroplast genome was 36.65%. A total of 133 functional genes were annotated, including 87 protein-coding genes (PCGs), 38 tRNA genes, and eight rRNA genes.

The complete chloroplast genomes of eight species from the Liliaceae family and two outgroups from the Chloranthaceae family were used to construct the maximum likelihood tree with 1000 bootstrap repeats (model: TVM + F+G4) by W-IQ-TREE (Trifinopoulos et al. 2016) after aligned by MAFFT 7 (Katoh and Standley 2013). The phylogenetic tree showed that *T. patens* was closely related to *Tulipa sylvestris* (Figure 1).

**Figure 1.**
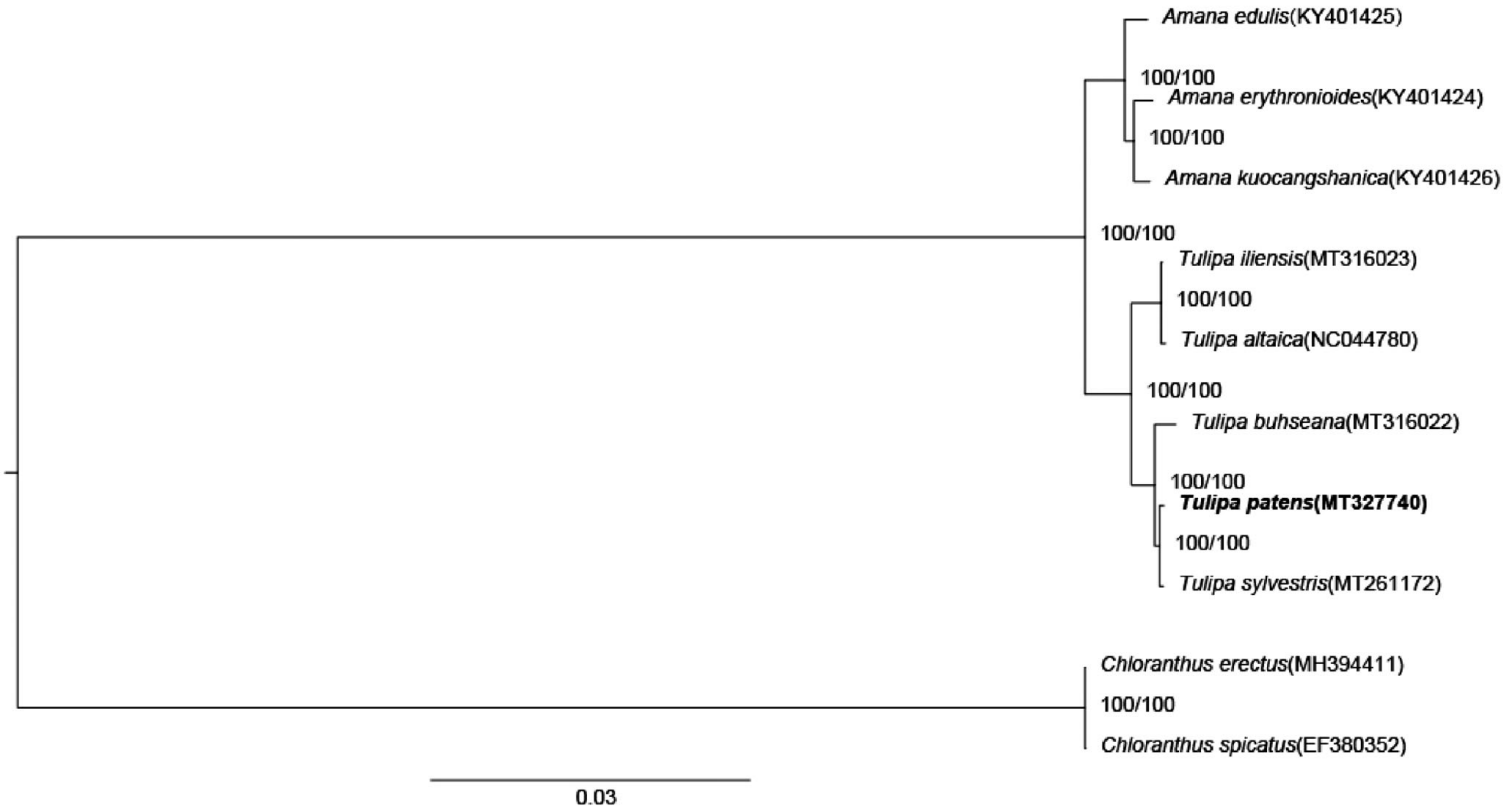
The maximum likelihood tree based on 10 complete chloroplast genome sequences. Support values written on the branches: SH-aLRT support (%)/ultrafast bootstrap support (%).

## Data Availability

The genome sequence data that support the findings of this study are openly available in GenBank of NCBI at (https://www.ncbi.nlm.nih.gov/) under the accession no. MT327740.The associated BioProject, SRA, and Bio-Sample numbers are PRJNA680208, SAMN16872356, and SRR13107963, respectively.
